# Predicting Hemodynamic Failure Development in PICU Using Machine Learning Techniques

**DOI:** 10.3390/diagnostics11071299

**Published:** 2021-07-20

**Authors:** Rosanna I. Comoretto, Danila Azzolina, Angela Amigoni, Giorgia Stoppa, Federica Todino, Andrea Wolfler, Dario Gregori, on behalf of the TIPNet Study Group

**Affiliations:** 1 Unit of Biostatistics, Epidemiology and Public Health, Department of Cardiac, Thoracic, Vascular Sciences and Public Health, University of Padova, 35131 Padova, Italy; rosanna.comoretto@unipd.it (R.I.C.); danila.azzolina@unife.it (D.A.); giorgia.stoppa@studenti.unipd.it (G.S.); federica.todino@studenti.unipd.it (F.T.); 2 Department of Medical Sciences, University of Ferrara, 44100 Ferrara, Italy; 3 Pediatric Intensive Care Unit, Department of Women’s and Children’s Health, University Hospital of Padua, Via Giustiniani 2, 35128 Padova, Italy; angela.amigoni@aopd.veneto.it; 4 Department of Anaesthesia, Gaslini Hospital, 16147 Genova, Italy; andreawolfler@gaslini.org

**Keywords:** machine learning techniques, hemodynamic failure, PICU, imbalance management, outcome prediction

## Abstract

The present work aims to identify the predictors of hemodynamic failure (HF) developed during pediatric intensive care unit (PICU) stay testing a set of machine learning techniques (MLTs), comparing their ability to predict the outcome of interest. The study involved patients admitted to PICUs between 2010 and 2020. Data were extracted from the Italian Network of Pediatric Intensive Care Units (TIPNet) registry. The algorithms considered were generalized linear model (GLM), recursive partition tree (RPART), random forest (RF), neural networks models, and extreme gradient boosting (XGB). Since the outcome is rare, upsampling and downsampling algorithms have been applied for imbalance control. For each approach, the main performance measures were reported. Among an overall sample of 29,494 subjects, only 399 developed HF during the PICU stay. The median age was about two years, and the male gender was the most prevalent. The XGB algorithm outperformed other MLTs in predicting HF development, with a median ROC measure of 0.780 (IQR 0.770–0.793). PIM 3, age, and base excess were found to be the strongest predictors of outcome. The present work provides insights for the prediction of HF development during PICU stay using machine-learning algorithms.

## 1. Introduction

Heart failure or hemodynamic failure (HF) in children is a rare progressive clinical and pathophysiological syndrome caused by cardiovascular and non-cardiovascular abnormalities that result in signs and symptoms including edema, respiratory distress, growth failure, and accompanied by circulatory, neurohormonal, and molecular dysregulation [1]. Causes, mechanisms, incidence, and prevalence of HF in children are more different from those in the adult population. Primary or secondary HF diagnosis in children could often be associated with congenital or vascular malformations or cardiomyopathies, in lower prevalence with infective endocarditis, myocarditis, rheumatic fever, pneumonia, anthracycline toxicity, and renal disorders [2]. In the last year, an increased rate of HF and myocardial dysfunction requiring pediatric intensive care unit (PICU) admission seemed to be largely spared due to the SARS-CoV−2 inflammatory multisystem syndrome [3]. Thus, the prognosis for children with HF is poor, and many cases progress to requiring emergency hospital management and need heart transplantation when drug therapy failed. However, in rare cases, pediatric HF could arise during hospitalization, especially in critically ill patients with severe baseline conditions, leading to a further worsening of the patients’ clinical status. Due to the rarity of the phenomenon, to our best knowledge, there are no studies about patients who developed HF during PICU stay.

Identifying patients at high risk of HF development would be helpful for clinicians since they could focus on managing such patients to prevent this severe condition and the potential worse outcome. However, as this condition could rarely arise during PICU stay, it is necessary (i) to use large registries that collect a vast amount of clinical information about PICU patients and (ii) to apply statistical methods that can both manage a high number of considered variables and account for the significant imbalance between the groups (low number of events vs. high number of non-events). Several methods have been employed to develop prediction models and risk scores in patients with cardiovascular diseases, from traditional statistical approaches to more advanced machine learning techniques (MLTs). In the adult population with HF, machine learning algorithms create risk scores estimating the likelihood of a heart failure diagnosis and the probability of outcomes such as all-cause mortality, cardiac death, and hospitalization [4,5,6,7]. MLTs are also increasingly used for hard outcome prediction in the clinical setting (e.g., in-hospital cardiac arrest) since they present several advantages over traditional methods and show promising performance and better power than existing prediction systems [8,9]. They help extricate complex relationships between covariates and outcome of interest, even though a low number of events have occurred with respect to the many variables to be tested.

Since no studies explore the issue of HF development in pediatric patients during the ICU length of stay, and since the overall prevalence of HF in the pediatric population has been reported to be 0.1% [2], the outcome to be estimated could be particularly imbalanced when compared with total ICU admissions. If this happens, in training an MLT model for risk stratification purposes, it could be biased for patients without HF (majority class) due to the imbalance in data and eventually increasing Type II errors. In such cases, MLT methods should be accompanied by imbalance management procedures to prevent them from becoming a null model, i.e., systematically tending to make incorrect predictions about the least represented HF class (minority class) [10].

Our study aims to identify predictors of new-onset HF during PICU stay testing a set of MLTs, comparing their ability to predict the outcome of interest by considering the main imbalance management techniques [11].

## 2. Materials and Methods

### 2.1. Data Source and Study Population

PICU admission data were extracted from the Italian Network of Pediatric Intensive Care Units (TIPNet) registry. TIPNet is a research network involving more than 20 Italian PICUs. We included patients admitted to 23 Italian PICUs in the period between 1 January 2010 and 31 December 2020. Only patients aged less than 18 years and for whom congenital heart disease or cardiovascular insufficiency was not the reason for PICU admission were considered. Data about clinical characteristics at admission were extracted and considered for the analyses, particularly those used for PIM 3 score calculation [12]. The outcome was represented by the development of hemodynamic failure (HF) during the PICU stay. All definitions of clinical conditions used in the present study are in relation to the need for admission in a pediatric intensive care setting and are specified in Table S1 (Supplementary Materials). 

Data are collected using a standardized electronic sheet (REDCap-platform, REDCap, TN, USA) [13] and anonymized at the moment of data extraction. All investigations were carried out following the Declaration of Helsinki. This study is part of a multiple research program based on TIPNet Registry, approved on 23 May 2014 by the Ethic Committee of Milano Area C—Niguarda Cà Granda Hospital (protocol number 269-052014).

### 2.2. Machine Learning Techniques

We constructed predictive models for hemodynamic failure during the PICU stay using the following approaches: generalized linear model (GLM), recursive partition tree (RPART), random forest (RF), neural networks models, and extreme gradient boosting (XGB).

- Generalized linear models (GLM) are frequently used to analyze binary data. We specified a complementary log-log link (clog log) given that an asymmetric link function is more appropriate when imbalanced data are analyzed [14]. We compared the GLM (clog log) model with machine learning techniques (MLTs) to see whether the ML approaches can deliver more remarkable results.
- RPART can be used for classification and regression tasks. Breiman proposed the classification and regression tree (CART), the best-known methodology for constructing decision trees (DT) [15]. The basic goal of a DT is to repeatedly decompose data into smaller subsets using a set of splitting rules until a specific stopping criterion is encountered. The key advantage of this method is that the tree structure is easily interpretable; however, this algorithm is prone to over-fitting [16].
- Random forest is a supervised ensemble learning method that builds a collection of decision trees obtained via bootstrap aggregation, resulting in a forest of trees to predict the outcome of interest. The RF model turns out to be less interpretable than DT but improves the robustness of predictions [17].
- In its simplest form, the neural network model is composed of an input layer, a hidden layer, and an output layer (three layers of neurons which are connected). The features extracted by the model represent the input layer and are used to predict an output. The nodes displayed in the input layer communicate with each node in the hidden layer, which is connected to an output layer. The purpose is to compute a weighted sum based on ‘neurons’ importance and to provide an output [18].
- A single-hidden-layer neural network (NNET) representing the simplest form of the neural network, in which there is only one layer of input nodes that send weighted inputs to a subsequent layer of receiving nodes has been considered for the computation.
- XGB is a decision-tree-based ensemble machine learning algorithm that can be used for classification or regression problems, increasing model accuracy. XGB uses a gradient boosting framework; the idea is to build trees sequentially in such a way as to ensure that the errors of the previous three are reduced; on the contrary, RF combines results at the end of the process training independently each classifier [19].

### 2.3. Imbalance Control and Missing Data Imputation Techniques

Since the outcome of interest is an infrequent event, a crucial numerical imbalance between the two groups is expected to be observed. Therefore, two different techniques for imbalance control have been applied: the downsampling and upsampling methods [20]. The downsampling technique (also called decimation), reduces the sampling rate of the most represented group [21]. The method randomly subset all the classes in the training set so that their class frequencies match the least prevalent (minority) class. A random sample of the majority class, having the same size as the minority class, has been performed and the procedure has been repeated within the resampling.

Conversely, the upsampling technique (or interpolation) increases the sampling rate of the less represented group. The procedure randomly samples (with replacement) the minority class to be the same size as the majority class [22].

### 2.4. Missing Data Imputation

The missing values’ imputations were performed by using a multivariate imputation by chained equations method (MICE). The method is based on a fully conditional specification, where a separate model imputes each incomplete variable. In addition, the predictive mean matching method has been considered for imputing numeric data; the logistic regression imputation for binary data, the polytomous regression imputation for unordered categorical data and the proportional odds model for ordinal variables [23]. Five imputations have been considered.

### 2.5. Model Training and Validation

The models were tuned within 100 bootstrap resampling iterations. A random search procedure was considered to identify the tuning parameters. This method sets up a grid of hyperparameter values and selects random combinations to train the model and score [24]. 

The model performances (ROC, accuracy, F statistics, sensitivity, and specificity) were computed within resamples and adjusted for over-optimism to internally validate the performance adjusting for overfitting [25]. The optimism adjusted bootstrap technique is as follows:

1. Fit a model on the whole data set.
2. Calculate the apparent performance of this model corresponding to the error that the model demonstrates on the original dataset.
3. Create 100 bootstrap resamples.
4. For every bootstrap resample, fit the model on that resample, calculate the apparent performance for this model on the bootstrap resample it was trained on, and find the apparent performance on the original dataset.
5. Calculate the optimism by performing the difference between the apparent performance obtained within the bootstrap resample and the apparent performance obtained on the original dataset.
6. Calculate the average optimism from all of the bootstrap samples.
7. The final performance is achieved by computing the difference between the original data apparent performance and the optimism measure [26].

### 2.6. Statistical Analysis

Descriptive statistics were reported as I quartile/median/III quartile for continuous variables and percentages (absolute numbers) for categorical variables. In addition, Wilcoxon-type tests were performed for the continuous variables and the Pearson chi-square test, or Fisher exact test, whichever is appropriate, for the categorical variables.

Sensitivity, specificity, accuracy, and the receiving operative characteristic curve (ROC) measures, with interquartile ranges (IQR), were used for model comparison and performance assessment. For the most promising MLT, the variable importance plot was reported together with the ROC curve and the median-balanced accuracy measure within the resampling.

The present analyses were performed using R Statistical Software 4.1.0 (Vienna, Austria) [27] with the rms [28], caret [29], and xgboost [30] packages.

## 3. Results

A sample of 31,453 subjects admitted to PICUs in the period between 1 January 2010 and 31 December 2020 was considered. Among these, 1959 were excluded: 251 were affected by congenital heart disease, 1262 had cardiovascular insufficiency at admission, and 446 presented both conditions. A final sample of 29,494 subjects was selected and, among these, only 399 developed hemodynamic failure during the PICU stay. Table 1 presents the subjects’ baseline characteristics according to the outcome development.

Overall, the median age of the subjects enrolled was about 2 years. The prevalence of the male gender was found to be higher than the female one (57% in the sample overall), even though it was found to be higher in subjects who did not develop hemodynamic failure during PICU stay (57% vs. 51%; *p*-value 0.020). Subjects who developed HF were significantly more likely to suffer from previous comorbidities, to be admitted with medical diagnosis, and to present organ failure at admission (*p*-value < 0.001), but less likely to be born for less than 7 days (*p*-value < 0.001). The prevalence of respiratory failure was found to be higher than for others. Furthermore, in subjects who developed the outcome, the elective admissions and those for surgery’s recovery were observed as significantly less (*p*-value < 0.001), but cases were more likely to be ventilated in the first hour after admission and presented a higher PIM 3 score (*p*-value < 0.001). No significant differences were detected in drug sedation conditions, in base excess values, and the prevalence of very high-risk or high-risk diagnoses.

### 3.1. MLTs Performance

Table 2 describes the different MLTs’ performances, using the original sample distribution or the two methods for imbalance control (downsampling and upsampling). Those algorithms and techniques were also applied to the original data without imputation of missing ones. 

**Figure 1:**
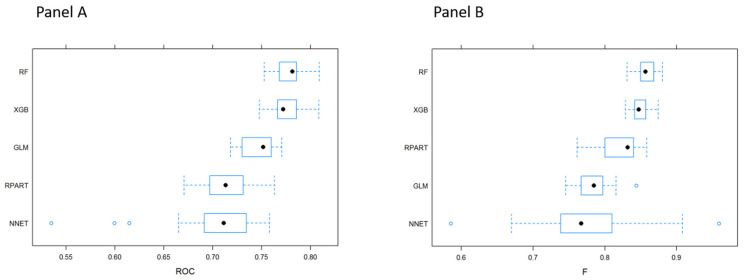
Comparison of the performance of the MLT algorithms. (Panel **A**) presents the ROC. (Panel **B**) presents the F-score measure.

Compared to the other sampling method, downsampling shows a better performance. After the imputation of missing data, random forest and XGB algorithms outperformed the other MLTs in predicting the development of HF during PICU stay (Table 2 and Figure 1). In particular, XGB presented a better ROC measure (median 0.780, IQR 0.770–0.793), and, looking at the values of sensitivity and specificity, it provided a better balance than the other techniques between these two measures.

Similar results were obtained considering only complete case data (Table S2, Supplementary Materials).

### 3.2. Variable Importance in Predicting the Outcome According to the Random Forest Model

**Figure 2:**
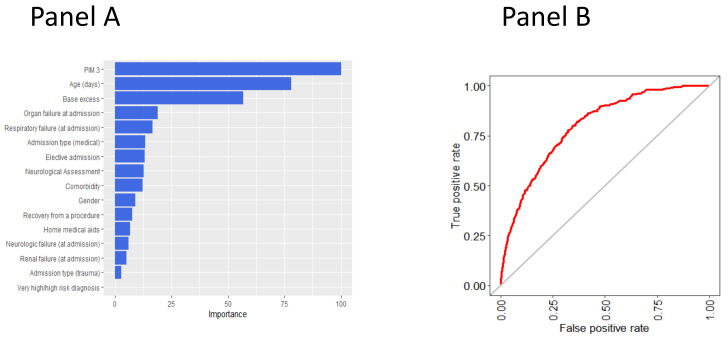
Random forest’s variable importance plot (Panel **A**) and ROC curve on the random forest predictions (Panel **B**).

Since random forest was found to be one of the two MLTs with the best performance, it was chosen to identify the predictors of in-PICU HF development. Figure 2 reports the plots of the variable importance measures according to the mean decrease accuracy. PIM 3 score and age, together with base excess, were found to be the strongest predictors of in-PICU HF development (Figure 2). Conversely, organ failure at admission, comorbidities and previous medical aids use, admission type, and the presence of very high-risk or high-risk diagnosis were found to provide only a small contribution in predicting the outcome.

A sensitivity analysis was performed reporting the variable importance measures across methods (Figure S3, Supplementary Materials). The importance measures agree across the best performing MLTs (RF and XGB) and NNET. Moreover, The PIM 3 is ranked across the first three leading predictors for all the considered MLTs.

## 4. Discussion

The present study aimed to compare the performance, in terms of accuracy level, of different MLTs in predicting HF development during PICU stay in patients enrolled in the TIPNet registry. Using the downsampling approach to control data imbalance, RF and XGB techniques were found to have the best performance in predicting the outcome. The average accuracy, sensitivity, and specificity of RF were 76.1%, 76.3%, and 62.3%, and 69.5%, 69.6%, and 71.3% for XGB. The ROC measures were 0.77 and 0.78, respectively.

From the clinical point of view, MLTs represent a promising opportunity to develop models able to predict hospital admissions/readmissions of HF patients [7] and hard in-hospital outcomes such as cardiac arrest, death, and the development of new severe clinical conditions. Churpek et al. conducted a multicenter study using machine learning methods for predicting clinical deterioration in the wards, including cardiac arrest, ICU transfer, and death. They reported that at 24 h before the occurrence, the RF and XGB models achieved the most accurate prediction, with AUCs of 0.80 and 0.79, respectively [31]. More recently, Yun et al. compared different machine learning algorithms’ abilities to predict in-hospital death of critically ill patients admitted to the surgical intensive care unit. They found that the decision tree showed higher classification results (AUC = 0.96) [32]. Moreover, Wu et al. found that the XGB algorithm was the most accurate compared to the other seven algorithms and showed promising discrimination for detecting in-hospital cardiac arrest, reporting accuracy of 88.9%, sensitivity of 73%, and F1 score of 80% [9].

To the best of our knowledge, there is no similar evidence on the pediatric population with HF, even if MLTs have also been primarily used in developing predictive models in critically ill children. For example, several studies used MLTs for early mortality prediction, the development of sepsis, or the need for pediatric intensive care unit transfer for newly hospitalized children [33,34,35]. However, no studies have investigated the development of a severe condition such as HF in patients admitted to PICU. This can certainly be justified by the fact that it is a sporadic event. In fact, in our cohort of almost 30,000 subjects, only 1.3% showed this outcome.

The present work showed an outperformance of random forest and XGB models compared to the other algorithms tested after a downsampling procedure for imbalance control. Comparing MLTs in predicting hard outcomes in adult ICUs setting (e.g., in-hospital mortality) is under debate but remains under-addressed in the pediatric ICU environment. However, the results agree with the literature concerning the mortality outcome prediction via MLT models in the ICU setting [36]. For example, the gradient boosting (GB) model showed the highest ROC (0.79 (0.77–0.80)) for the 30-day mortality prediction in mechanically ventilated patients, followed by the random forest model (0.78 (0.76–0.80)). Other studies conducted to predict the ICU mortality outcome in COVID−19 patients confirmed that the random forest model, followed by the GB predictive tool, outperformed the other MLTs [37]. Furthermore, the XGB model performs better, in comparison with the other MLTs, in predicting the adverse outcome of critically ill influenza patients admitted to the ICU [38]. Generally, the random forest, GB, and XGB methods are demonstrated to make more accurate predictions than an individual MLT model since they are ensemble methods combining the predictions from multiple learners [16].

Most medical datasets are not balanced in their class labels [39]. In addition, the greater part of MLTs tends not to perform well in predicting the minority underrepresented classes without adopting imbalance management procedures [40]. The most common methods to handle imbalance are based on downsampling and upsampling algorithms [41]. This research revealed that the downsampling method handles the imbalance between classes more successfully than the upsampling one, achieving a more real trade-off between predictive sensitivity and specificity. Moreover, these findings agree with the literature indicating a more effective performance for the downsampling procedure compared to the upsampling [41].

On the other hand, the upsampling methods may involve more computational time efforts during the training procedure and are more prone to overfitting issues [40]. The downsampling procedure outperforms the upsampling in terms of computational efforts and memory storage [41]. Moreover, Drummond and Holte [42] demonstrated that the downsampling procedures yield a better minority class prediction performance in comparison with the upsampling procedure.

Interestingly, the variables found to contribute to in-hospital mortality were PIM 3 score and age. At the same time, the organ failures, comorbidities, and admission types played a minor role in the HF development prediction. However, it is worth pointing out that it is difficult to compare results from the literature since this outcome has never been explored before. Notably, we found that the most important variables in the random forest model have also been shown to be important predictors in prior research in pediatric settings [12]. Recently, Lee et al. developed a machine learning model for predicting pediatric mortality in the early stages of intensive care unit admission and compared it with PIM 3 score [33]. Interestingly, they found that the random forest model performed better than PIM 3 score in predicting mortality. Furthermore, as in our results, base excess was among the more significant clinical variables associated with the outcome. However, in the present study, laboratory data and instrumental parameters have not been used in prediction models, but it would be interesting to explore a different set of baseline variables for predicting such an outcome.

Since the number of subjects experiencing the outcome is very limited, the main limitation of the present study lies in the fact that it is difficult to fragment and specify more effectively the subjects’ characteristics. Moreover, the quality of care might change during the study period; therefore, results may not be generalized for patients in more recent years. However, on the other hand, using an extensive multicenter database allowed us to still be able to capture the phenomenon, which otherwise would have been impossible using monocentric data.

## 5. Conclusions

Present findings suggest that MLTs may be a promising opportunity to predict HF development in pediatric patients during ICU stay by exploiting baseline information collected by a multicenter registry. However, further research is needed to improve their accuracy level and to better evaluate their usefulness in clinical practice.

## Figures and Tables

**Table 1:** Subjects’ baseline characteristics according to outcome development (HF). Data are I quartile/median/III quartile for continuous variables and percentages (absolute numbers) for categorical ones.

Characteristics	*N*	No HF (*N* = 29,095)	HF (*N* = 399)	Combined (*N* = 29,494)	*p*-Value
Age (days)	29,494	133/754/2766	132/881/2874	133/755/2768	0.402
Gender: Male	29,494	57% (16,559)	51% (204)	57% (16,763)	0.020
Female		43% (12,536)	49% (195)	43% (12,731)	
Newborn: Yes	29,485	6% (1665)	2% (7)	6% (1672)	<0.001
No		94% (27,421)	98% (392)	94% (27,813)	
Comorbidity: Yes	27,931	45% (12,451)	59% (235)	45% (12,686)	<0.001
No		55% (15,081)	41% (164)	55% (15,245)	
Home medical aids: Yes	26,153	7% (1886)	5% (20)	7% (1906)	0.114
No		93% (23,883)	95% (364)	93% (24,247)	
Admission type: Surgical	27,921	45% (12,322)	17% (68)	44% (12,390)	<0.001
Medical		50% (13,771)	76% (304)	50% (14,075)	
Trauma		5% (1429)	7% (27)	5% (1456)	
Organ failure at admission: Yes	27,458	58% (15,759)	87% (349)	59% (16,108)	<0.001
No		42% (11,300)	13% (50)	41% (11,350)	
Respiratory failure (at admission): Yes	29,494	48% (14,010)	76% (305)	49% (14,315)	<0.001
No		52% (15,085)	24% (94)	51% (15,179)	
Neurologic failure (at admission): Yes	29,494	9% (2538)	19% (77)	9% (2615)	<0.001
No		91% (26,557)	81% (322)	91% (26,879)	
Renal failure (at admission): Yes	29,494	3% (823)	12% (47)	3% (870)	<0.001
No		97% (28,272)	88% (352)	97% (28,624)	
Drug sedation: Yes	27,454	32% (8686)	31% (124)	32% (8810)	0.663
No		68% (18,369)	69% (275)	68% (18,644)	
Ventilation in the first hour: Yes	26,393	48% (12,378)	66% (252)	48% (12,630)	<0.001
No		52% (13,636)	34% (127)	52% (13,763)	
Base excess	26,368	−3.0/0.0/0.0	−4.7/0.0/1.2	−3.0/0.0/0.0	0.143
Elective admission: Yes	26,390	44% (11,351)	19% (71)	43% (11,422)	<0.001
No		56% (14,660)	81% (308)	57% (14,968)	
Recovery from a procedure: Yes	26,394	47% (12,159)	28% (108)	46% (12,267)	<0.001
No		53% (13,855)	72% (272)	54% (14,127)	
PIM 3	26,280	0.28/0.77/2.20	1.02/3.25/8.12	0.28/0.79/2.27	<0.001
Very high/high risk diagnosis: Yes	29,494	89% (25,764)	88% (351)	89% (26,115)	0.717
No		11% (3331)	12% (48)	11% (3379)	

Abbreviations: HF, hemodynamic failure; PIM, Pediatric Index of Mortality.

**Table 2:** Comparison of the performance of the MLT algorithms (after missing data imputation). Measures are reported as I quartile/median/III quartile.

Technique	Sampling Method	Sensitivity	Specificity	Accuracy	ROC
**GLM**	Original sampling	1/1/1	0/0/0	0.985/0.986/0.987	0.756/0.769/0.782
	Downsampling	0.672/0.690/0.705	0.651/0.681/0.714	0.672/0.690/0.703	0.745/0.756/0.771
	Upsampling	0.659/0.671/0.675	0.688/0.699/0.735	0.659/0.671/0.675	0.761/0.769/0.776
**RPART**	Original sampling	0.997/0.998/0.999	0/0.0123/0.020	0.984/0.985/0.986	0.608/0.643/0.709
	Downsampling	0.672/0.715/0.725	0.566/0.596/0.669	0.673/0.714/0.723	0.704/0.719/0.724
	Upsampling	0.940/0.943/0.946	0.171/0.194/0.218	0.930/0.933/0.936	0.559/0.568/0.580
**NNET**	Original sampling	0.999/0.999/1	0/0/0	0.985/0.986/0.986	0.711/0.722/0.741
	Downsampling	0.585/0.625/0.685	0.690/0.762/0.791	0.588/0.627/0.685	0.694/0.720/0.745
	Upsampling	0.638/0.669/0.700	0.675/0.717/0.757	0.639/0.670/0.700	0.715/0.725/0.742
**RF**	Original sampling	1/1/1	0/0/0	0.986/0.986/0.987	0.739/0.748/0.757
	Downsampling	0.756/0.763/0.771	0.587/0.623/0.640	0.754/0.761/0.768	0.757/0.769/0.776
	Upsampling	0.999/1/1	0/0.007/0.014	0.985/0.985/0.986	0.731/0.739/0.744
**XGB**	Original sampling	1/1/1	0/0/0.006	0.986/0.986/0.987	0.750/0.758/0.769
	Downsampling	0.689/0.696/0.714	0.673/0.713/0.750	0.689/0.695/0.714	0.770/0.780/0.793
	Upsampling	0.962/0.964/0.966	0.156/0.171/0.180	0.952/0.953/0.954	0.718/0.723/0.733

Abbreviations: GLM, generalized linear models; RPART, recursive partition tree; NNET, neural networks; RF, random forest; XGB, extreme gradient boosting; ROC, receiving operative characteristic.

## Data Availability

The data presented in this study are available upon request from the corresponding author.

## Supplementary Materials

The following are available online at https://www.mdpi.com/article/10.3390/diagnostics11071299/s1, Table S1: Definition of outcome and clinical conditions considered in the study; Table S2: Comparison of the performance of the MLT algorithms (complete cases-without imputation); Figure S1: Random forest’s variable importance plot for the XGB, GLM, NNET, RPART, RF methods.
