# Supplementary material for: Predicting Hemodynamic Failure Development in PICU Using Machine Learning Techniques
Source: Diagnostics (Basel). 2021 Jul 20;11(7):1299. doi: 10.3390/diagnostics11071299 (PMC8303657; doi:10.3390/diagnostics11071299)
Supplement: Supplementary file 1 [file diagnostics-11-01299-s001.zip › Table S1.pdf]

Table S1. Definition of outcome and clinical conditions adopted in the TIPNet database.

Condition	Definition
**Hemodynamic failure (outcome)**	Need to use catecholamines
**Respiratory failure**	Need for oxygen therapy or need for ventilation
**Renal failure**	Oliguria or impaired kidney function (based on normal thresholds depending on age)
**Neurological failure**	P/U level at AVPU scale; Glasgow Coma Scale score < 9
**Coagulopathy***	Impaired coagulation or platelet reduction, platelet count <= 80,000/mm^3 or a 50% reduction in value from the highest of the previous three days or INR> = 2
**Liver failure***	Total plasma bilirubin > 4 mg/dl in the absence of haemolysis, neonatal hyper-bilirubin or primary liver disease or ALT greater than twice the age limit
**Organ failure**	The presence of at least one condition among those reported above

Abbreviations: AVPU, alert, verbal, pain, unresponsive.
* These deficiencies, being very low in our sample, were not considered individually but fall into the 'Organ failure' macro-category.
