# Supplementary material for: Predicting Hemodynamic Failure Development in PICU Using Machine Learning Techniques
Source: Diagnostics (Basel). 2021 Jul 20;11(7):1299. doi: 10.3390/diagnostics11071299 (PMC8303657; doi:10.3390/diagnostics11071299)
Supplement: Supplementary file 1 [file diagnostics-11-01299-s001.zip › Table S2.pdf]

Table S2. Comparison of the performance of the MLT algorithms (complete cases - without imputation). Measures are reported as I quartile/Median/III quartile.

Technique	Sampling Method	Sensitivity	Specificity	Accuracy	ROC
**GLM**	Original sampling	1 / 1 / 1	0 / 0 / 0	0.985 / 0.985 / 0.986	0.738 / 0.750 / 0.758
	Downsampling	0.624 / 0.648 / 0.666	0.671 / 0.706 / 0.735	0.626 / 0.649 / 0.666	0.730 / 0.752 / 0.760
	Upsampling	0.636 / 0.644 / 0.667	0.677 / 0.690 / 0.715	0.637 / 0.644 / 0.667	0.738 / 0.746 / 0.760
**RPART**	Original sampling	0.995 / 0.996 / 0.997	0.007 / 0.014 / 0.021	0.980 / 0.981 / 0.983	0.667 / 0.683 / 0.708
	Downsampling	0.670 / 0.716 / 0.729	0.559 / 0.604 / 0.646	0.670/ 0.715 / 0.727	0.697 / 0.713 / 0.731
	Upsampling	0.974 / 0.975 / 0.977	0.058 / 0.077 / 0.091	0.962 / 0.963 / 0.965	0.516 / 0.525 / 0.531
**NNET**	Original sampling	1 / 1 / 1	0 / 0 / 0	0.985 / 0.986 / 0.986	0.754 / 0.762 / 0.769
	Downsampling	0.587 / 0.625 / 0.683	0.68 / 0.725 / 0.773	0.590 / 0.626 / 0.684	0.691 / 0.711 / 0.734
	Upsampling	0.609 / 0.672 / 0.700	0.647 / 0.706 / 0.791	0.612 / 0.672 / 0.699	0.701 / 0.733 / 0.744
**RF**	Original sampling	0.999 / 0.999 / 0.999	0 / 0 / 0.007	0.985 / 0.986 / 0.986	0.710 / 0.715 / 0.732
	Downsampling	0.742 / 0.752 / 0.772	0.604 / 0.634 / 0.671	0.741 / 0.751 / 0.769	0.768 / 0.781 / 0.786
	Upsampling	0.998 / 0.998 / 0.999	0.007 / 0.013 / 0.015	0.984 / 0.985 / 0.985	0.713 / 0.723 / 0.731
**XGB**	Original sampling	0.998 / 0.998 / 0.998	0.007 / 0.019 / 0.027	0.984 / 0.985 / 0.985	0.725 / 0.737 / 0.745
	Downsampling	0.730 / 0.738 / 0.754	0.616 / 0.658 / 0.678	0.728 / 0.737 / 0.752	0.766 / 0.772 / 0.786
	Upsampling	0.990 / 0.991 / 0.991	0.044 / 0.060 / 0.071	0.977 / 0.978 / 0.979	0.732 / 0.743 / 0.750

Abbreviations: GLM, Generalized Linear Models; RPART, Recursive Partition Tree; NNET, Neural Networks; RF, Random Forest; XGB, Extreme Gradient Boosting; ROC, Receiving Operative Characteristic.
