# Supplementary figures and images for: Predicting Hemodynamic Failure Development in PICU Using Machine Learning Techniques
Source: Diagnostics (Basel). 2021 Jul 20;11(7):1299. doi: 10.3390/diagnostics11071299 (PMC8303657; doi:10.3390/diagnostics11071299)

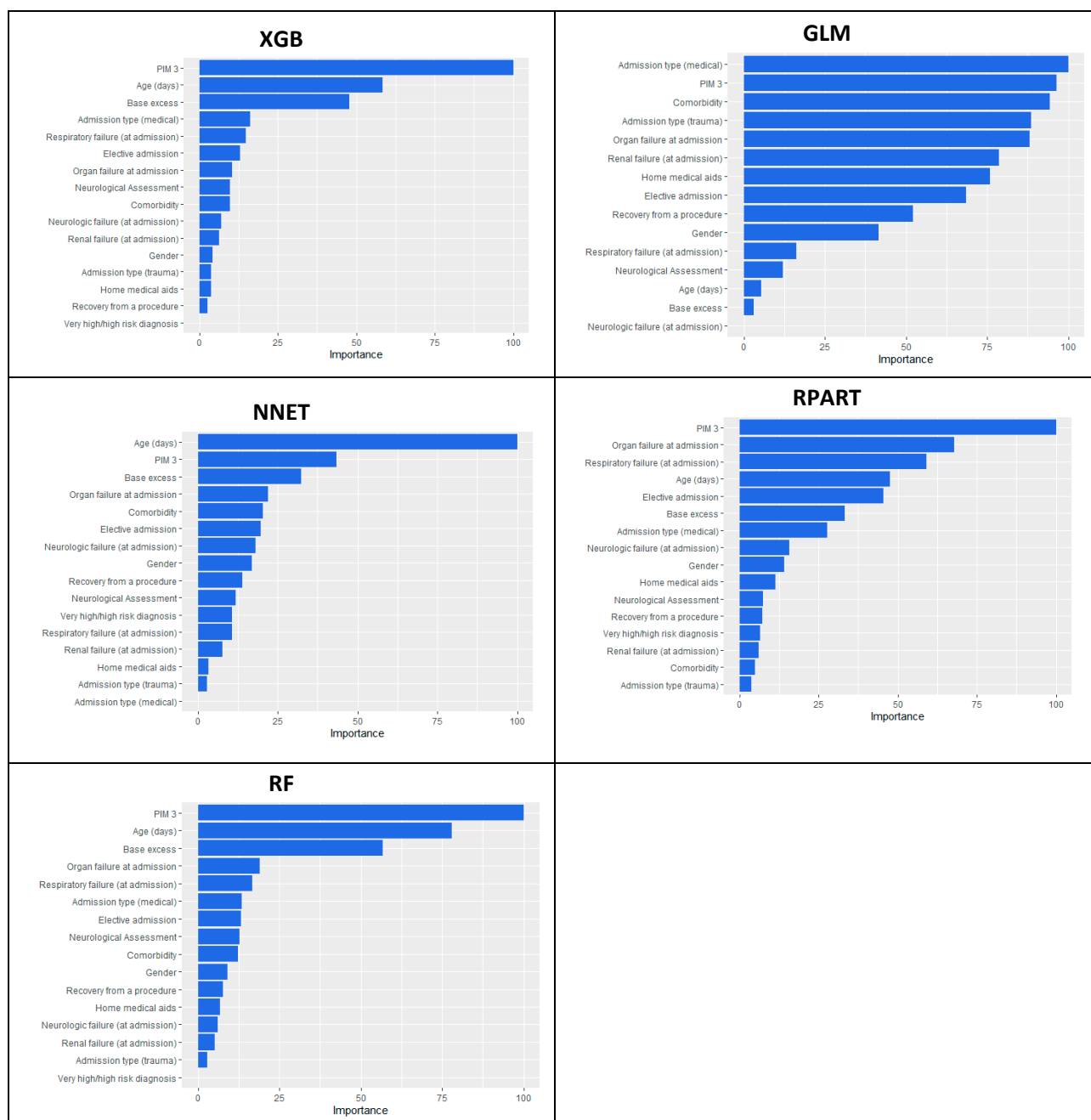

**Figure S1.** Random Forest's variable importance plot for the XGB, GLM, NNET, RPART, RF methods.

Supplement: Supplementary file 1 [file diagnostics-11-01299-s001.zip › Figure S1.pdf]
